# Text mining for clinical support

**DOI:** 10.5195/jmla.2019.758

**Published:** 2019-10-01

**Authors:** Jonathan Hartmann, Linda Van Keuren

**Affiliations:** Director of Clinical Integration Services & Data Discovery, Dahlgren Memorial Library, Georgetown University Medical Center, 3900 Reservoir Road Northwest, Washington, DC 20057-1420, jth52@georgetown.edu; Assistant Dean for Resources & Access Management, Dahlgren Memorial Library, Georgetown University Medical Center, 3900 Reservoir Road Northwest, Washington, DC 20057-1420, lav30@georgetown.edu

## Abstract

**Background:**

In 2013, the Dahlgren Memorial Library (DML) at the Georgetown University Medical Center began using text mining software to enable its clinical informationists to quickly retrieve specific, relevant information from MEDLINE abstracts while on patient rounds.

**Description:**

In 2013, DML licensed the use of the Linguamatics I2E text-mining program, and DML’s clinical informationist began using it to text mine MEDLINE abstracts on patient rounds. In 2015, DML installed I2E on a server at Georgetown and negotiated with Elsevier to obtain the right to download and text mine the full text of clinical journals in ScienceDirect to support clinical decision making. In 2016, the license agreements for the *New England Journal of Medicine* and the *BMJ* platform were modified to allow text mining. In 2018, PubMed Central open access content was added to the Linguamatics license.

**Results:**

DML’s informationists found that they were able to quickly find useful information that was not retrievable by traditional methods, and clinicians reported the information was valuable.

**Conclusion:**

The ability to text mine MEDLINE abstracts and selected journal articles on patient rounds has allowed DML’s clinical informationists to quickly search large amounts of medical literature that can be used to answer physicians’ clinical questions. DML plans to acquire additional journal articles from selected publishers in the future, which should increase the usefulness of the project.

The Dahlgren Memorial Library (DML) is the graduate health and life sciences research library for the 6,500 full-time equivalent (FTE) students, faculty, researchers, physicians, nurses, and staff at the Georgetown University Medical Center. Since the early 2000s, the library has focused on building an online-only collection, thus providing a 99% online collection today. DML has also provided clinical informationist services to the MedStar Georgetown University Hospital for the past ten years. These services include participation in daily patient rounds [1].

In 2013, a representative of Linguamatics contacted DML’s clinical informationist and asked if he would be interested in using Linguamatics’ I2E text-mining software through its OnDemand service. Text mining is a natural language processing (NLP) technology that can extract facts, relationships, and assertions that would otherwise remain buried in masses of text. This process differs from traditional keyword searching, which retrieves entire documents that then need to be reviewed to find relevant information. The OnDemand service provides access to the full MEDLINE database, to which Linguamatics’ server provides access.

The clinical informationist tested I2E and found he could quickly retrieve detailed information from MEDLINE abstracts without having to read through numerous abstracts as would have often been necessary using a traditional search engine. This is because a snippet of relevant text from each abstract is immediately displayed in the search results, enabling the user to quickly find the desired information [2]. As a result, DML decided to license I2E to enable its clinical informationist to quickly retrieve specific, relevant information from MEDLINE abstracts while on patient rounds. This partnership was the first of its kind for both organizations.

Because DML’s clinical informationist used an iPad on patient rounds and I2E was designed for computers and was not mobile compliant, he worked with Linguamatics staff to develop a web interface. Queries for common search categories were developed and added to the web interface so that queries would not have to be developed from scratch on rounds. Initially, five queries were created based on the informationist’ s experience of common types of questions. The queries were then tested on rounds, and based on this testing, the queries were refined and nineteen additional queries were developed.[2] The queries were designed so that the informationist could simply add specific terms to the query and I2E would automatically select appropriate subject headings.

In 2015, DML became aware that Elsevier would allow text mining of their content for research purposes. Therefore, DML negotiated the right to download and text mine the full text of the medical journals in the Elsevier ScienceDirect platform. DML then worked with Georgetown University’s Information Services (UIS) department to use Elsevier’s application programming interfaces (APIs) to download the articles onto the UIS server. Also, to text mine the downloaded articles, the Enterprise version of I2E had to be installed on the UIS server. There was an additional cost for the Enterprise version of I2E and the local server space. Although I2E was now installed locally, DML’s informationist was still able to use the OnDemand service to search MEDLINE.

The project grew substantially over the next few years. In 2016, DML obtained permission to text mine current content from the *New England Journal of Medicine* and *BMJ Journals*. Unlike the arrangement with Elsevier where an API is used to download content, these articles are provided via weekly file transfer protocol (ftp) feeds from the publishers to the local server. The following year, a second informationist was added to the license and trained to use the software. Expansion continued in 2018 with the addition of over one million open access articles in PubMed Central. As is the case with MEDLINE, these articles are accessible via Linguamatics server through the OnDemand service.

The ability to text mine MEDLINE abstracts and full-text journal articles on patient rounds has allowed DML’s clinical informationists to quickly search large amounts of medical literature that would otherwise be unsearchable, thus allowing them to provide additional answers to physicians’ clinical questions, for three reasons. First, text mining allows the entire text of articles to be searched, not just titles and abstracts as in traditional keyword searching. Second, snippets of relevant text are immediately displayed in the search results, instead of titles and abstracts as in traditional searching. Third, unlike subject or keyword searching, text mining uses NLP algorithms, which allow it to recognize similar concepts even if they have been expressed in very different ways or with different spellings. DML’s clinical informationists have found Linguamatics I2E to be a valuable supplemental tool for searches where traditional search functionality is not able to quickly retrieve needed information.

In the future, DML plans to acquire additional journal articles from selected publishers that allow text mining of their content, which should increase the usefulness of the project.

**Jonathan Hartmann, MLS**, jth52@georgetown.edu, https://orcid.org/0000-0001-6629-9947, Director of Clinical Integration Services & Data Discovery, Dahlgren Memorial Library, Georgetown University Medical Center, 3900 Reservoir Road Northwest, Washington, DC 20057-1420

**Linda Van Keuren, MLS, AHIP**, lav30@georgetown.edu, Assistant Dean for Resources & Access Management, Dahlgren Memorial Library, Georgetown University Medical Center, 3900 Reservoir Road Northwest, Washington, DC 20057-1420

## References

[1] Twombly R (2014). iPad in hand, librarian joins rounds [Internet].

[2] Hartmann J, Singh G (2016). Evolution of I2E to improve patient care [Internet].

